# Healthy Effects of Plant Polyphenols: Molecular Mechanisms

**DOI:** 10.3390/ijms21041250

**Published:** 2020-02-13

**Authors:** Manuela Leri, Maria Scuto, Maria Laura Ontario, Vittorio Calabrese, Edward J. Calabrese, Monica Bucciantini, Massimo Stefani

**Affiliations:** 1 Department of Experimental and Clinical Biomedical Sciences “Mario Serio”, University of Florence, Viale Morgagni 50, 50134 Florence, Italy; manuela.leri@unifi.it (M.L.); monica.bucciantini@unifi.it (M.B.); massimo.stefani@unifi.it (M.S.); 2 Department of Neuroscience, Psychology, Drug Research and Child Health, University of Firenze, 50139 Florence, Italy; 3 Department of Biomedical and Biotechnological Sciences, University of Catania, Torre Biologica, Via Santa Sofia, 97-95125 Catania, Italy; mary-amir@hotmail.it (M.S.); Marialaura.ontario@ontariosrl.it (M.L.O.); vittorio.calabrese@unict.it (V.C.); 4 Department of Environmental Health Sciences, School of Public Health and Health Science, University of Massachusetts, Amherst, MA 01003, USA

**Keywords:** plant polyphenols, hormesis, autophagy, Mediterranean diet, olive oil, curcumin, resveratrol, oleuropein, hydroxytyrosol, epigallocathechin, epigenetics

## Abstract

The increasing extension in life expectancy of human beings in developed countries is accompanied by a progressively greater rate of degenerative diseases associated with lifestyle and aging, most of which are still waiting for effective, not merely symptomatic, therapies. Accordingly, at present, the recommendations aimed at reducing the prevalence of these conditions in the population are limited to a safer lifestyle including physical/mental exercise, a reduced caloric intake, and a proper diet in a convivial environment. The claimed health benefits of the Mediterranean and Asian diets have been confirmed in many clinical trials and epidemiological surveys. These diets are characterized by several features, including low meat consumption, the intake of oils instead of fats as lipid sources, moderate amounts of red wine, and significant amounts of fresh fruit and vegetables. In particular, the latter have attracted popular and scientific attention for their content, though in reduced amounts, of a number of molecules increasingly investigated for their healthy properties. Among the latter, plant polyphenols have raised remarkable interest in the scientific community; in fact, several clinical trials have confirmed that many health benefits of the Mediterranean/Asian diets can be traced back to the presence of significant amounts of these molecules, even though, in some cases, contradictory results have been reported, which highlights the need for further investigation. In light of the results of these trials, recent research has sought to provide information on the biochemical, molecular, epigenetic, and cell biology modifications by plant polyphenols in cell, organismal, animal, and human models of cancer, metabolic, and neurodegenerative pathologies, notably Alzheimer’s and Parkinson disease. The findings reported in the last decade are starting to help to decipher the complex relations between plant polyphenols and cell homeostatic systems including metabolic and redox equilibrium, proteostasis, and the inflammatory response, establishing an increasingly solid molecular basis for the healthy effects of these molecules. Taken together, the data currently available, though still incomplete, are providing a rationale for the possible use of natural polyphenols, or their molecular scaffolds, as nutraceuticals to contrast aging and to combat many associated pathologies.

## 1. Introduction

Human beings in advanced countries are experiencing an increasing extension in life expectancy, yet at the cost of a progressively greater prevalence of a number of diseases associated with lifestyle and age. The latter include widespread pathologies such as cancer, metabolic disease such as type 2 diabetes mellitus (T2DM), hepatitis and the metabolic syndrome (MetS), cardiovascular diseases (CVDs), and amyloid diseases, both systemic and neurodegenerative, particularly Alzheimer’s (AD) and Parkinson (PD) diseases. Among the latter pathologies, amyloid diseases, both the sporadic and the early onset familial forms, are substantially intractable. In particular, most sporadic amyloidoses are characterized by slow progression, often covering several decades, and their clinical signs appear only at mid- or old age, when they are irreversible due to conspicuous cell loss. It is then evident that the absence of fully reliable early diagnostic tools and of resolutive therapies, particularly in the case of amyloid diseases, calls for prevention in the role of best approach to face these pathological conditions.

Following the considerations reported above, it is not surprising that research has progressively shifted from a “cure” to “prevention” focus, in addition to the “drugs”, and the “lifestyle” concept, including “diet”, and hence “food”. Many epidemiological and observational studies support the idea that the Mediterranean diet (MD) and the Asian diet are associated with “safe” aging, with a reduced prevalence of diseases associated with aging including CVDs, cancer, and cognitive decline [1]. The MD is the result of a complex socio-economic development of the Mediterranean populations in which agricultural, social, territorial, and environmental practices represent a heritage intimately associated with their culture and lifestyle that has been passed through several generations over many centuries. The great value of the MD and the associated lifestyle has been recognized by the “Organizzazione delle Nazioni Unite per l’Educazione, la Scienza e la Cultura“ (UNESCO) that, since November 2010, has inscribed the MD in the list of the intangible cultural heritage of humanity (https/ich.unesco.org/). Recently, the “Mediterranean Diet Foundation Expert Group” has proposed a new MD pyramid [2]; the latter, besides confirming the importance of the daily assumption of substantial amounts of vegetables, fruits, legumes, grains, nuts, seeds, and, in particular, of a moderate intake of red wine and of extra virgin olive oil (EVOO) as the main lipid source, also emphasizes a healthy lifestyle where, in addition to caloric restriction (CR), frugality, moderation, adequate rest, conviviality, culinary activities, and physical exercise play an important role.

A key aspect of the MD is the high assumption of phytonutrients (particularly vitamins and plant phenols) that, by themselves, interfere with multiple signaling pathways involved in protein homeostasis, DNA repair, metabolism regulation, and antioxidant defenses that, in most cases, recall CR [3,4]. Many available data and population studies suggest that greater adherence to the MD reduces metabolic pathologies and aging-associated deterioration; these data have been further supported by a number of clinical trials carried out by administering foods enriched in specific polyphenols to patients at cardiovascular or neurodegeneration risk, suffering from metabolic diseases, or affected by some forms of cancers (see below). Biophenols are found in many foods of plant origin that are key components of the MD and the Asian diet; these include red wine, EVOO, green tea, spices, berries, and aromatic herbs that ensure a continuous daily moderate intake of these substances during lifespan. Hence, the presence of substantial amounts of polyphenols in the diet may provide a rationale for the results of the large number of population studies and clinical trials supporting the idea that an association does exist between dietary practices, their content in polyphenols, and a significant reduction of the incidence of many aging-associated diseases [5].

The studies carried out during the last decade have highlighted the fact that, in the case of amyloid diseases and several other aging-associated pathologies, plant polyphenols do not simply affect a single step of the pathogenesis of these diseases such as the aggregation path of the involved protein/peptide, the inflammatory response, the alteration of the proteostatic network, and/or the oxidative stress; rather, the biological and functional features of their positive outcomes result from multi-target effects that restore several altered homeostatic systems in the cells and tissues where impaired metabolism or protein/peptide aggregation occur. Moreover, these molecules share key chemical features that, at least in part, explain why most of them, although chemically different, induce similar effects. Recent research is increasingly shedding light in the biochemical, cellular, and epigenetic modifications produced by a number of investigated plant polyphenols, with special emphasis on the olive ones, and the ensuing modulation of key cellular processes such as metabolic/energetic/redox homeostasis, proteostasis, signaling, and control of oxidative stress and of gene expression. These data are providing the knowledge needed to fully understand the beneficial effects of the MD and the Asian diet and the importance of plant polyphenols for human wellness and as possible tools useful in disease prevention and, possibly, therapy.

Here, we summarize the results of the studies on a number of plant polyphenols, with particular emphasis on those found in the olive leaves and drupes and in the EVOO, particularly oleuropein aglycone (OLE) and hydroxytyrosol (HT). We also provide data on the population surveys and clinical trials supporting the beneficial effects of the MD, its content in fruit, vegetables, herbs, and EVOO, and the intake of these molecules. We also focus the main molecular mechanisms by which plant polyphenols ensure protection against a number of age-related pathologies, in light of the recently proposed hormetic theory (see below); the molecular determinants underlying such protection both in biological models such as cultured cells and animals and the most recent advances towards their possible use in prevention and, possibly, as co-adjuvants in clinical treatment of neurodegenerative diseases, CVDs, cancer, T2DM, and the MetS are also discussed.

## 2. Polyphenols: Important Players of the Mediterranean/Asian Diets

Natural biophenols are a wide group of molecules (over 8000 described so far) found only in the plant kingdom; their molecules display one or more aromatic rings carrying one or more hydroxyl groups; these molecules display remarkable antioxidant power and are produced as secondary metabolites by the plant for protection against the attack by bacteria, fungi, and insects (phytoalexins) [1]. Plant polyphenols include non-flavonoids or flavonoids; the latter are further divided into flavonols, flavononols, flavones, anthocyanins, procyanidins, phenolic acids, stilbenes, and tannins depending on the number of hydroxyls in the molecule and on the nature and the position of other substituents [6]. The natural phenols most studied for their healthy properties ar curcumin, a phenolic acid found in the rhizome of *Curcuma longa Linn* (family Zingiberaceae) and a component of the curry; epigallocathechins, notably epigallocatechin-3-gallate (EGCG), the flavanol found in green tea; resveratrol (3,5,4′-trihydroxy-*trans*-stilbene), a stilbene found in grapes and in red wine; quercetin and myricetin, flavonols present in tea, onions, cocoa, red wine, and in *Ginkgo biloba* extracts. Many other plant phenols or their metabolic derivatives have also been investigated, yet with variable results; these include tannic, ellagic, ferulic, nordihydroguaiaretic and caffeic acid, morin, rutin, apigenin, baicalein, kaempferol, fisetin, luteolin, rottlerin, malvidin, piceatannol, and silibinin.

More recently, particular interest has been dedicated to the polyphenols present abundantly in the leaves and in the ripening fruits of the olive tree (*Olea europaea*); including flavonols, lignans iridoids, and their glycosides. Iridoids are monoterpenes containing a cyclopentane ring fused to a six-atom heterocycle with an oxygen atom, whereas the molecules where the cyclopentane ring is interrupted are indicated as secoiridoids. The most abundant polyphenols found in the EVOO include the secoiridoids oleuropein and ligstroside, both in the glycated and in the aglycone forms, and their main metabolites: The phenolic alcohols tyrosol (p-hydroxyphenylethanol, p-HPEA) and hydroxytyrosol (3,4-dihydroxyphenylethanol, 3,4-DHPEA), oleacein, and oleocanthal. Oleuropein confers the bitter taste to the olive leaves, drupes, and EVOO, whereas oleocanthal produces the burning sensation in the back of the throat when consuming EVOO [7,8]. Phenolic concentration in plants depends on several variables; in the olive tree, and hence in the EVOO, these include (i) olive cultivar and stage of ripening [9]; (ii) environmental cues (altitude, cultivation practices, meteorological factors, and irrigation); (iii) technological aspects such as extraction conditions (temperature and others) and systems to separate oil from olive pastes; and (iv) storage conditions and time, due to spontaneous oxidation, and deposition of suspended water droplets with their content of polyphenols [10]. At the best, polyphenol content in the EVOO can exceed 60 mg/100 g.

Plant polyphenols have been considered for their remarkable antioxidant properties; however, their effects go well beyond this property. In fact, plant polyphenols have been shown to possess beneficial effects against aggregation of peptides/proteins into amyloid assemblies, a process involved in several amyloid diseases, particularly T2DM, AD, and PD, thus reducing the load of intra- or extracellular deposits [1]. T2DM is characterized by aggregates of the peptide amylin in insulin-secreting pancreatic β-cells; AD results from the extracellular amyloid aggregation of the Aβ peptide (mostly Aβ_1–42_) in senile and neuritic plaques and the intracellular aggregation into neurofibrillary tangles of tau protein; these aggregates are found in specific brain areas, notably the hippocampus and the pre-frontal cortex. PD results mostly from the aggregation of α-synuclein into intracellular Lewy bodies found in the neurons of the mesencephalic *substantia nigra*. Actually, olive polyphenols, notably OLE and its main metabolite, HT, have been reported to be effective against amyloid aggregation and the ensuing pathologic effects in T2DM [11,12], AD [13,14,15,16,17,18,19] and, possibly, in PD [20,21] and other amyloidoses. Plant polyphenols, including those enriched in EVOO, have also been reported to reduce insulin resistance and to improve impaired glucose homeostasis, two main signs of T2DM. Considering that increasing evidence supports a strong link between diabetes (mainly T2DM) and neurodegeneration associated with AD [22,23], it is conceivable that many plant polyphenols can interfere with both pathologies at similar molecular levels.

**Figure 1:**
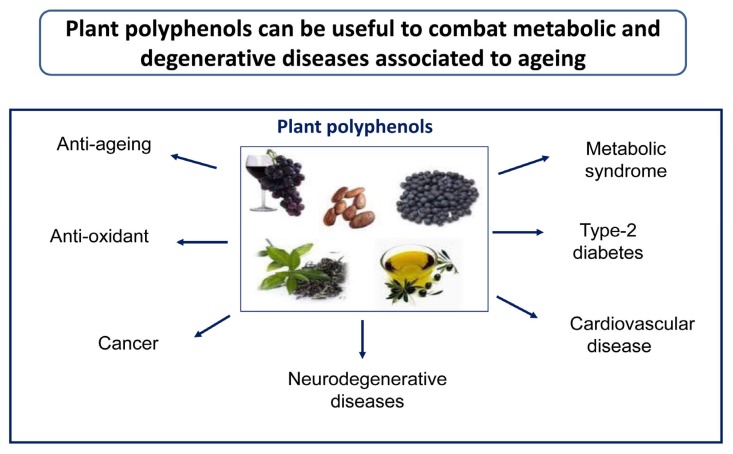
Plant polyphenols can be useful to prevent/combat a number of lifestyle-, metabolic-, and aging-associated pathologies.

The beneficial effects of plant preparations and derivatives, including, in the case of olive, oil and leaf extracts, have already been known for the last couple of centuries and have been scientifically investigated over the recent several decades; these researches have progressively led to a focus on the multi-target activity and health properties of plant polyphenols, including the anti-amyloid aggregation, antioxidant, antimicrobial, antihypertensive, hypoglycemic, and vasodilator effects. The antioxidant power has been shown to involve modulation of oxidative pathways [2], direct action on enzymes, proteins, receptors, and several types of signaling paths [3,4], as well as the interference with epigenetic modifications of chromatin [24] (see below). The clinical significance of the beneficial properties of plant polyphenols was first reported in 1950 [25], leading to the inclusion in the European Pharmacopoeia (Ph. Eur.) of the 80% alcoholic extract of olive leaves, containing oleuropein, HT, caffeic acid, tyrosol, apigenin, and verbascoside [26,27]. Biophenols can also be used to develop new drugs useful to combat chronic inflammatory conditions, the risk of thrombosis, CVD-related states such as atherosclerosis [28], cancer [29], also in combination with anti-cancer drugs [30], as well as to reduce amyloid deposition associated with T2DM and aging-related states such as neurodegeneration [1,31] (Figure 1). Finally, the molecular scaffolds of plant polyphenols are also investigated to develop new molecules potentially exploitable in disease prevention and therapy [32].

The beneficial effects of olive and other plant polyphenols can be hindered by the reduced bioavailability of the latter following reduced intestinal absorption and their rapid biotransformation in the organism; in addition, chemical modifications of many polyphenols by gut microbiota further reduces their bioavailability and biological efficacy [33]. Several strategies to overcome reduced bioavailability have been proposed, including encapsulation within nanoparticles [34], self-microemulsifying formulations [35], and others (reviewed in [36]). Yet, several studies have shown that many plant polyphenols, including EVOO polyphenols, are absorbed, although in reduced amount, by humans and distributed, in part, to organs and tissues before completion of their secondary metabolism, degradation, and excretion [37,38,39]. Moreover, some OLE metabolites, notably HT, arising mainly from acid hydrolysis in the stomach, have been found in the brain of TgCRND8 mice and rats after an acute oral administration of either OLE or HT, which supports the ability of OLE and some of its derivatives, including HT, to cross the blood–brain barrier [40,41]. In particular, convincing evidence indicates that HT is dose-dependently bioavailable; it is substantially (40%) absorbed and reaches maximum concentration in plasma after 5–30 min from EVOO ingestion, with a calculated total bioavailability around 10% due to metabolic modifications in the gut and in the organism through phase II conjugation reactions [42]. Thus, in spite of reduced information on many aspects of their pharmacokinetics, pharmacodynamics, and metabolic modifications by gut microbiota and in the organism, oral assumption of plant polyphenols results in partial absorption and distribution to the whole organism (reviewed in [43]).

## 3. The Beneficial Effects of Plant Polyphenols Are Supported by Population Studies and Clinical Trials

A wealth of currently available data arising from population studies suggest that greater adherence to the MD improves healthy aging by reducing the risk of cardiovascular diseases (CVDs), metabolic pathologies, cancer, and aging-associated mental deterioration; in particular, it enhances cognitive function and reduces both the risk of developing mild cognitive impairment and of its conversion to AD [44,45]. The beneficial effects of the MD have been related to the presence, in the latter, of many polyphenols, particularly those enriched in fruits and vegetables, in red wine, and in EVOO (reviewed in [1,43]). The importance of the content of polyphenols (EGCG, OLE, HT, resveratrol, curcumin, quercetin, morin, myricetin, and many others) in the MD has been confirmed by many population studies and clinical trials.

Over 60 years ago, the first large-population study on the diet–health relation, the “Seven Countries Study”, reported quite a low rate of cardiovascular mortality in Crete farmers, though in the presence of a large intake of lipids, and related that finding to their MD (reviewed in [46]). Some years later, other studies confirmed and extended these results. The “Lyon Diet Heart Study” further supported the MD–myocardial infarction relation, leading to the conclusion that the MD may delay considerably, up to 46 months, the rate of recurrence [47]. The “Three-City Study”, conducted on 7000 elderly subjects, is one of the most cited studies on the protection by olive oil against neurodegeneration and cognitive decline. The results of the study, published in 2009, showed a lower rate of cognitive deficit in subjects whose diet was characterized by the presence of variable amounts of olive oil with respect to the subjects using other lipids [48].

More recently, the randomized “PREvención con DIeta MEDiterránea” (PREDIMED) and PREDIMED-NAVARRA studies have been conducted in Spain on subjects at high cardiovascular risk (477 and 522 participants, respectively) [49,50,51]. In particular, the PREDIMED-NAVARRA study was originally carried out on high-cardiovascular-risk asymptomatic subjects (187 people, 67 years mean age) on MD supplemented with olive oil, with nuts, or at control diet to test the effect of the two types of MD on the progression of subclinical carotid atherosclerosis. The results showed significant improvement in mean intima-media thickness with regression of carotid atherosclerosis after one year in people on either MD [52]. The PREDIMED study was a primary prevention trial originally designed to test the long-term effects of the MD on the incidence of CVDs in people at high cardiovascular risk; the trial suggested that olive oil consumption matches reduced risk and mortality for CVDs [53,54]. The same cohort was also checked for cognition, which was significantly improved in people following an EVOO-enriched MD [49,51]. The study also investigated breast cancer incidence in the same cohort, leading to the conclusion that the risk of developing cancer was reduced by 68% in the EVOO group [55]. Finally, data from a subsample (*n* = 990) of the PREDIMED study showed that the MD significantly decreased low-density lipoproteins (LDL) oxidation only when it was enriched in EVOO with medium-high phenolic content [56].

More recently, a cross-sectional sampling population study was carried out among Spanish people in 100 health centers, where 4572 individuals aged > 18 years, representative of the Spanish population, were analyzed on the basis of their use of olive or sunflower oil. According to the study, the intake of olive oil resulted as beneficial against several cardiovascular risk factors, particularly in obese people with a sedentary lifestyle, impaired glucose tolerance, and insulin resistance [57]. Another study carried out in the USA investigated the relation between intake of olive oil and incidence of T2DM; the study followed 59,930 women (35–65 years) for 22 years from the Nurses’ Health Study and 85,157 women (26–45 years) from the NHS II with no diabetes, CVDs, or cancer at baseline. The results indicated that a slightly lower risk of T2DM was associated with higher olive oil intake, whereas an increased risk was associated with the assumption of other lipids in place of olive oil [58].

A very recent crossover study carried out with 25 healthy subjects randomly distributed to a MD without EVOO or supplemented with 10 g EVOO/day concluded that EVOO reduces post-prandial levels of LDL-cholesterol and glucose, further supporting the positive effects of the MD against atherosclerosis [59]. Moreover, a small crossover trial (Trial number: ISRCTN09220811) was conducted in the context of the “EUROLIVE” study with 200 participants who, after a two-week washout, assumed 25 mL of olive oil with different phenolic content three times in a day for three weeks. The results indicated a linear decrease of the oxidative stress markers ratio and of the total cholesterol/HDL-cholesterol matching the content of polyphenols in olive oil [60]. A subset of the same trial suggested protection against atherosclerosis [61]. Another subset of the study confirmed that the phenolic content in olive oil increases human HDL functionality by favoring HDL-mediated efflux of cholesterol from macrophages [62]. In another multicenter trial in Spain, 7447 participants (55–80 years) at high cardiovascular risk, but with no CVD at enrollment, were assigned to a MD supplemented with EVOO, a MD supplemented with mixed nuts, or a control diet (with reduced fat). The authors reported that the incidence of major cardiovascular events was lower in the participants on an EVOO- or nut-supplemented MD than in those at a diet with reduced fat. [63].

In 2010, a study with 20 patients with the MetS showed that the acute intake of olive oil fortified with olive polyphenols resulted in positive changes of the postprandial expression of disease-involved genes in mononuclear cells from peripheral blood [64]. Studies were also carried out to determine the effects of an acute administration of olive oil, particularly in the case of protection against postprandial hyperlipidemia and the associated inflammation. Another research conducted on 20 obese subjects showed that oils rich in phenols reduced postprandial inflammation by lowering the levels of pro-inflammatory cytokines and by activating nuclear factor κB (NF-κB) [65]. Some other studies reported a remarkable delay in living day activities decline together with improved cognition, psychopathology, and quality of life of the involved people [66,67,68]. Recently, a study carried out on 62 institutionalized elderly humans fed with EVOO showed a significant improvement of their redox status, with positive effects on lipid profiles and total antioxidant capacity, significantly increased activity of catalase in erythrocytes, and decreased activity of superoxide dismutase and glutathione peroxidase [69]. Finally, a systematic review evaluated the effect of the intake of olive oil with high or low polyphenol content on CVDs risk factors, as emerged in a number of clinical trials; the study concluded that polyphenol-rich olive oil confers some benefits resulting in reduction of CVD-risk, even though the authors claimed that more prolonged studies in non-Mediterranean populations were needed to better validate the data [70].

The reported observational studies, population surveys, and randomized controlled trials on the MD–cardiovascular health relation have been critically reviewed very recently. The study included the five most comprehensive meta-analyses published in the 2014–2018 period together with additional prospective studies for a total of 45 reports. The authors concluded that higher conformity to the traditional MD matches better cardiovascular health outcomes in terms of reduced rates of coronary heart disease, ischemic stroke, and CVD [71]. In spite of the existence of remarkable differences among the investigated populations, the results of other studies substantially confirmed the studies reported above and also highlighted a beneficial effect of the MD against cognitive deterioration, particularly in AD and PD (reviewed in [72]) and against cancer. In particular, a study carried out in Australia and New Zealand (ACTRN12613000602729) showed that a cause–effect relationship does exist between MD and aging-dependent impairment of cognition [73]; these results were confirmed by a study carried out with a cohort of aged people, the “Prevenciòn con Dieta Mediterrànea Nutrition Intervention Trial” (ISRCTN35739639), where a significant improvement of cognitive behavior following MD supplementation with olive oil or nuts was reported [74].

Several clinical studies, mostly carried out with a limited number of patients, sought to unravel the role played by specific polyphenols in the MD against the same pathological conditions, yet with variable results. The effects of curcumin against AD have been investigated in four clinical trials (reviewed in [75]); two studies reported that no significant differences were found between placebo- and curcumin-treated groups where cognition, plasma, and cerebrospinal fluid (CSF) biomarkers were concerned [76,77]; these data were confirmed by other, more recent, trials with no solid results on protection by lipidated curcumin against neurodegeneration in AD [78]. Over 244 clinical trials, with an additional 27 clinical trials currently ongoing, investigated the healthy effects, safety, and pharmacokinetics of resveratrol. Resveratrol administration gave positive results in patients suffering from diabetes mellitus, obesity, MetS, hypertension, stroke, CVDs, colorectal and breast cancer, multiple myeloma, AD, inflammatory diseases, and others, as summarized in a very recent review [79]. Other clinical trials reported a positive modulation by resveratrol of enzyme systems involved in carcinogen activation and detoxification together with some improvement of cerebral blood flow (reviewed in [80]). Recently, a clinical trial with *trans*-resveratrol was conducted on 28 obese men with the MetS and insulin resistance in the metabolic unit of the Rockefeller University Hospital. The subjects randomly received 1 g resveratrol orally twice a day for 35 days while consuming a Western-style diet. The subjects were tested for glucose tolerance, energy expenditure at rest, blood pressure, and abdominal adipose tissue. The results indicated that resveratrol altered glucose homeostasis only slightly except in a small group of Caucasians, where it improved insulin resistance and glucose homeostasis [81]. The beneficial effects of green tea, particularly of epigallocatechins, have been largely investigated even in clinical trials addressing their anti-cancer properties; however, the reported data do not permit to draw solid conclusions indicating a decreased risk of breast cancer by green tea consumption, due to the high heterogeneity of case-control and cohort studies (reviewed in [82]). Recently it has been suggested that green tea and/or green tea extracts inhibit the progression of cardiac amyloidoses [83]; however, currently, reduced information is available in humans on the protection against amyloidoses and amyloid-associated neurodegeneration specifically attributable to EGCG. Finally, *Ginkgo biloba* extracts were administered in several clinical trials carried out in aged people, both healthy, demented, or after ischemic stroke. Some of these studies yielded positive outcomes for what protection against cognitive decline was concerned (reviewed in [84]). The positive effects of plant polyphenols against breast, colon, and colorectal cancer, as emerged from clinical trials, preclinical studies, and primary research, have been recently reviewed critically [85,86,87,88].

Clinical studies involving specific olive polyphenols are still in their infancy. In 2012, the efficacy of an olive leaf extract as a hypoglycemic agent both in diabetic rats and in humans with improved glucose homeostasis, reduction of fasting plasma levels of insulin and glycated hemoglobin was reported [89]. Soon after, the antidiabetic properties of this polyphenol were further supported by the results of a randomized, placebo-controlled, crossover trial carried out with 46 middle-aged overweight men; the study confirmed the improvement of glucose homeostasis with a reduction of glycated hemoglobin and fasting plasma insulin levels in the enrolled patients [90]. More recently, the results of a randomized controlled clinical trial carried out with 60 pre-hypertensive males fed for six weeks with an olive leaf extract containing 136 mg of OLE and 6 mg of HT agreed with previous data, indicating the hypotensive and lipid-lowering effects associated with OLE intake [91]. Olive leaf extracts also improve human vascular function, as indicated by a randomized, double-blind, placeb-controlled, crossover trial carried out with nine healthy males and nine healthy females on a normal diet or on a diet supplemented with 51 mg of OLE and 10 mg of HT (four-week washout) [92]. A recent study aimed at identifying modifications of gene and miRNAs expression following intake of high- or low-polyphenols EVOO was carried out in 12 healthy subjects and 12 patients with the MetS by using microarray and real-time PCR (RT-PCR). Gene and miRNAs expression analysis in PBMCs prior of after EVOO administration showed that high-polyphenols EVOO induced modifications of the transcriptome in the investigated cells; in particular, a number of pathways associated with pathophysiological alterations of CVDs, metabolic diseases, and cancer were modulated. In healthy subjects, the acute intake of EVOO enriched in polyphenols not only resulted in improved glycaemia and decreased insulin resistance, but also modulated the transcription of genes and miRNAs involved in metabolism, inflammation, and cancer, inducing a less severe inflammatory phenotype in peripheral blood mononuclear cells (PBMCs); weaker effects were described in patients with the MetS as well as in healthy subjects administered with low-polyphenol EVOO [93]. Comparable results were reported in a study where the effects of HT on the expression of inflammation-related genes were investigated by RT-PCR in Simpson Golabi Behmel Syndrome (SGBS) adipocytes in terms of mRNA and microRNA levels. The results indicated that HT modulates the expression of adipocyte genes through oxidative stress reduction and inhibition of NF-κB, thus hindering macrophage recruitment and reducing the deregulation of signaling pathways involved in pathologies associated with obesity, with overall improvement of the inflammatory state of the adipose tissue [94]. Another recent randomized controlled trial was carried out with 22 healthy volunteers at post-prandial assumption of 25 g of polyphenols-enriched EVOO. After 2 h from the intake, a significant improvement of the redox, inflammatory, and metabolic status was detected, with decrease of oxidized LDL, malonyldialdehyde, triglicerides, and visceral adiposity index, resulting from upregulation of superoxide dismutase 1, catalase, and upstream transcription factor 1 [95]. Finally, a systematic review of randomized controlled trials aimed at focusing the state of the art of the relation between daily polyphenol consumption, circulating CVD biomarkers, and iron level has been undertaken very recently. The reported results involved seven studies that agreed with the inclusion criteria, and indicated that the intake of polyphenols results in a significant improvement of the inflammatory biomarkers and lipid profile without any interference with iron levels [96].

Many other trials carried out with a number of other plant biophenols, in most cases, gave positive outcomes against the same pathologies as those reported above. Taken together, the clinical trials data appear encouraging, yet in the absence of definitive results on the effective benefits of the various investigated polyphenols against a number of pathologies, and on the effective doses at which these effects are fully reached. In particular, large clinical trials aimed at evaluating effective protection by specific polyphenols against the most widespread neurodegenerative diseases are still lacking. In conclusion, clinical trials and population studies support consistent and effective protection by the MD and Asian diet, particularly associated with the prolonged intake of their polyphenols, against the insurgence of aging-associated pathologies, notably neurodegeneration, CVDs and metabolic diseases, and, possibly, cancer (Table 1). In particular, taking into consideration the positive results of the large number of clinical and population studies supporting the healthy power of olive polyphenols, in November 2011, the European EFSA authorized a claim stating the benefits of the assumption of olive oil polyphenols, suggesting a daily intake of 5 mg (http://www.efsa.europa.eu/).

## 4. Hormesis: A New General Concept Supporting Polyphenol Benefits

**Figure 2:**
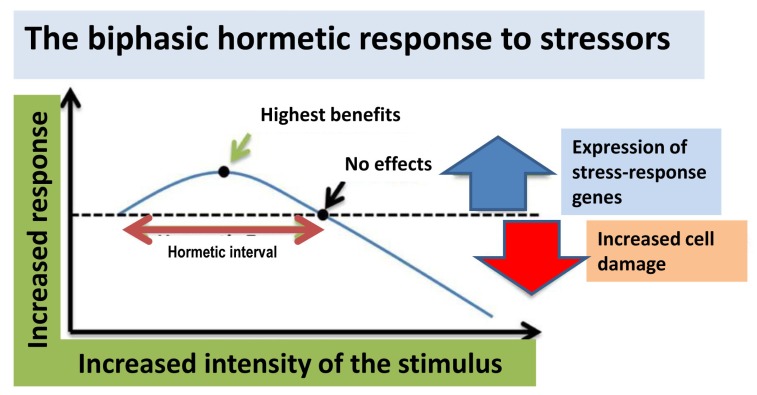
Hormesis describes a biphasic dose-response feature to stressful stimuli.

Hormesis is defined as any process in a cell or an organism characterized by a biphasic response to the exposure to increasing amounts of a stressing condition or substance (stressor) [98]. Generally, a favorable biological response to low exposure to any stressor is found within the hormetic zone, whereas cell damage occurs at higher doses (Figure 2). Plant polyphenols are produced by plants to defend themselves against pests such as bacteria, fungi, and insects, to which they are toxic. Such toxicity is not seen in higher animals at the maximal doses so far used experimentally, reaching 10 g/day in the case of resveratrol. However, these polyphenols act as stressors to the exposed animal cells, with the result of activating various cell defense systems aimed at controlling the redox environment, the proteostatic and metabolic homeostasis, organelle turnover, the inflammatory response, and others, thus making cells more resistant to subsequent toxic stimuli, particularly in aged people.

**Figure 3:**
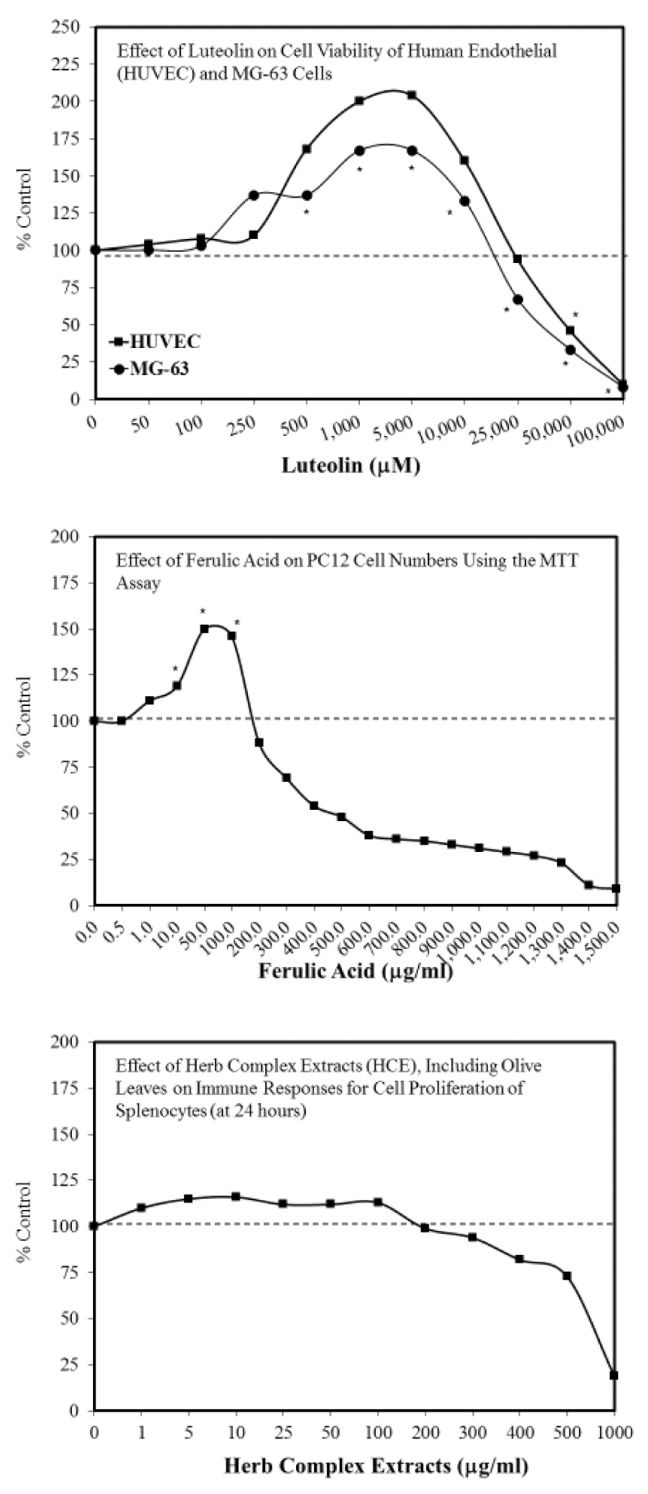
Upper panel: Effect of luteolin on cell viability of human endothelial (HUBEC) and MG-63 cells (adapted from [104,105]). Middle panel: Effect of ferulic acid on PC12 cell numbers using the MTT assay (adapted from [106]). Lower panel: Effect of Herb Complex Extracts (HCE), including Olive Leaves on Immune Responses for Cell Proliferation of Splenocytes (at 24 h) (adapted from [107]). All graph data are reported respect to control rapresented with dotted line.

In addition to many substances, including alcohol and many plant molecules, notably polyphenols, most stressors include physical agents and lifestyle such as physical exercise, CR, and, possibly, mild oxidative stress, while the role of low doses of ionizing radiations is debated. When assessed over a broad dose–response range, all these agents display a hormetic-biphasic dose–response relation. The biphasic dose–response results in low dose stimulation and high dose inhibition. Typically, the highest stimulatory responses are only about 30%–60% greater than in the control group [99,100,101]. It is within the low-dose stimulatory range where health benefits associated with polyphenols occur. While maximum stimulatory responses have been shown to be highly consistent in the 30%–60% stimulatory range, the hormetic stimulatory dose range can be quite valuable, depending on the agent, model, and endpoint agent, with the stimulatory dose zone typically being in the 5–50-fold dose range below the threshold. However, it is not uncommon for the stimulatory dose range to be in the 100- to 1000-fold dose range. These quantitative features can have important public health implications since a primary goal is to optimize the beneficial response while avoiding toxicity at higher doses. A more extensive assessment of the occurrence of hormesis [102] and its mechanistic foundations [103] is provided in these references. Several examples of hormetic dose responses for constituents of olive oil/olive leaf extracts are provided in [104,105,106,107] and in Figure 3.

## 5. Plant Polyphenols Rescue Altered Homeostatic Systems in Cells

### 5.1. Redox Homeostasis and the Inflammatory Response

Emerging research has recently focused on biological relevance for cell protection in many degenerative diseases and for neuroprotection in several neurodegenerative disorders, particularly AD and PD, of the redox homeostasis elicited by plant polyphenols through the activation of vitagene signaling pathways. The latter involves redox sensitive genes such as the Hsp70, heme-oxygenase-1 (HO-1), thioredoxin/thioredoxin reductase, and sirtuins system [108]. All these cytoprotective genes can be transcriptionally modulated by the nuclear factor erythroid 2–related factor 2 (Nrf2) as a part of the electrophile counterattack, defined phase 2 response. Nrf2 is a key transcription effector for the activation of wide range of cytoprotective genes (>500). The Nrf2 activity induces a mild stress response, providing a healthy physiological steady state and extending lifespan in different cells and animal models. On the other hand, a chronic long-term Nrf2 stimulation may lead to pathophysiological events, therefore the Nrf2 signaling can be considered as a hormetic-like pathway [109,110]. Increasing evidences show that plant polyphenols activate the phase 2 response leading to the expression of various Nrf2-dependent antioxidant vitagenes. These effects represent a powerful instrument supporting redox homeostasis under stressful conditions [111] and support the assumptions that the helpful properties of polyphenols carry out through adaptive stress response vitagenes. Within this context, both heme oxygenase-1 (HO-1) and Hsp70 have received considerable attention for their recognized antioxidative function needed to maintain cell homeostasis [112]. Moreover, the well-known principle of hormesis may be applied also to HO-1. In fact, although several studies have shown the crucial role of HO-1 activity against oxidative and nitrosative stress [113,114], excessive upregulation of HO-1 system may be deleterious for cells, because of accumulation of its by-products such as carbon monoxide (CO), iron, and the bilirubin precursor, biliverdin [115]; in light of the concept of hormesis, it is reasonable to look at these by-products of HO1 activity in terms of their positive effects obtained at very low concentrations.

As reported by Naviaux and coworkers [116], the hormetic response is activated when chemical-physical or biological hazards surpass the cellular capacity for homeostasis. The ensuing disruption of homeostasis induces a cascade of deleterious molecular changes in cells involving electron flow, oxygen consumption, and redox potential, that result in alterations of cellular structures and processes, including membrane fluidity, bioenergetics, protein folding, and misfolding. An increasing body of evidence shows that most chronic diseases arise from the biological response to a stress factor, not from the initial injury, or from the agent of the injury itself. The initial components of this cascade elicit the release of ATP, ADP, metabolic intermediates of Krebs cycle, oxygen, and reactive oxygen species (ROS) that is sustained by purinergic signaling pathway [116]. The anti-inflammatory and resilience phenotypes arise from these initial adaptive processes eventually mediate protection from a variety of potential injuries.

Dietary plant polyphenols that act as powerful antioxidants may block/abolish the anti-inflammatory phenotype that results from a redox regulatory signaling pathway [117,118,119,120]. More recent findings suggest that the biphasic dose responses of hormetic type are common effects of plant polyphenols. Interestingly, plant polyphenols are considered as a “preventive treatment of disease” inducing biological effects with important therapeutic applications to in vitro and in vivo models through the activation of adaptive responses [121]. Currently, increasing evidence suggests that plant polyphenols such as curcumin, sulforaphane, resveratrol, HT, and OLE may offer beneficial effects acting in a hormetic-like manner by activating adaptive stress-response pathways and making the hormesis concept fully applicable to the field of nutrition. Moreover, it has been considered that plant polyphenols may be protective through hormetic processes that involve the stress-activated “vitagenes” [117,122].

Chronic neuroinflammation is a prominent feature shared by several neurodegenerative diseases, such as AD and PD. Microglial activation, the hallmark of brain neuroinflammation, results in the production of highly pro-inflammatory cytokines (i.e., TNF-α, IL-β, prostaglandin E2, cyclooxygenases, and iNOS through the modulation of signal transduction pathways), ROS, and NO, leading to cellular modifications including mitochondrial dysfunction, impaired energy metabolism, altered redox homeostasis, lipid peroxidation, DNA fragmentation, neuronal inflammation, and cell death; recently, these alterations have been associated with the pathogenesis of neurological disorders [123,124]. Inflammasomes are multiprotein signaling complexes that regulate cells of the innate immune system, mainly microglial cells in the brain. Notably, a considerable amount of information has provided evidence on the existence of inflammasome-mediated inflammatory pathways in neurological diseases. In particular, the NLR family, pyrin domain-containing-3 (NLRP3) inflammasome has been shown to play a pathogenic role in the development of neuroinflammatory disorders, such as AD [125] and PD [126].

In vivo and in vitro studies have highlighted a direct relationship between inflammasome activation and AD pathogenesis, suggesting that inflammasome inhibition represents a potential therapeutic approach for AD treatment. This concept was confirmed in APP/PS1mice (transgenic mice with chronic deposition of Aβ) where a deficiency of NLRP3 inflammasome and caspase-1 activity were found [127]. Moreover, in these mice models, the production of microglia with phenotype M2 induced by NLRP3 inflammasome deficiency was observed, resulting in reduced deposition of Aβ [127]. In addition, in humans, enhanced caspase-1 activation was found mainly in the hippocampal area of human brain tissue of patients with AD, [127]. Moreover, in an AD mouse model, administration of cathepsin-B (produced by microglia after Aβ phagocytosis) inhibitors significantly decreased the load of amyloid plaques in the mouse brain tissue and resulted in substantial improvement in memory deficit. In PD patient brains, the inflammasome pathway can potentially be activated by oxidative stress and by insoluble α-synuclein aggregates [128]. The pathophysiological link between inflammasome responses and Aβ-plaque spreading indicates that pharmacological targeting of inflammasomes may represent a novel, potential treatment strategy for AD and PD.

In light of the data reported above, the activities of plant polyphenols against neuroinflammation appear to target activated microglia resulting in low-level production of pro-inflammatory molecules induced by the NRLP3 inflammasome. In particular, many recent data suggest the key role played by phenolic components of EVOO in counteracting protein misfolding and proteotoxicity, with a particular emphasis on the mechanisms leading to the onset and progression of AD and PD, including APP processing, Aβ peptide and tau amyloid aggregation, autophagy impairment, disruption of redox homeostasis, α-synuclein neurotoxicity, and neuroinflammation [48,72]. Additionally, several data indicate that OLE interferes with APP processing [129] and with the amyloid aggregation of Aβ and tau protein, escaping the growth of toxic Aβ oligomers both in vitro [17,19,130,131], in *C. elegans* [132], and in TgCRND8 mice, a transgenic model of Aβ deposition [40]. In accordance with these data, a recent study has reported that rats fed with OLE display significant improvement of cognitive performance; in fact, OLE diet decreases the apoptosis and oxidative stress levels and prevents the impairment of spatial learning and memory resulting from morphine-induced neurotoxicity to the hippocampus [133].

It is noteworthy that HT, a dopamine metabolite, is present in the brain [134]. Monoamine oxidase (MAO) catalyzes oxidative deamination of dopamine in a toxic metabolite, DOPAL (3,4-dihydroxyphenylaldehyde), that can be oxidized by aldehyde dehydrogenase (ALDH) to DOPAC (3,4-dihydroxyphenylacetic acid), the major metabolite of dopamine in the brainor may be reduced to HT by alcohol dehydrogenase (ADH). At the same time, DOPAC reductase can transform DOPAC in HT [135]. DOPAL is a highly reactive metabolite, suggesting that it might be a neurotoxic dopamine metabolite with a role in the pathogenesis of PD [136,137].

HT removes soluble oligomeric Aβ_1–42_ plus ibotenic acid-induced neurobehavioral dysfunction following intracerebroventricular injection of Aβ_1–42_ [130]. In hippocampal neurons, HT treatment rescues, to a significant extent, negatively altered spatial reference and working memory induced by Aβ, an effect associated with reduced activation of death kinases such as JNK and p38-MAPK, while concomitantly increasing the survival-signaling pathways such as ERK-MAPK/RSK2, PI3K/Akt1, and JAK2/STAT3 [130].

In human trials, a higher adherence to the MD was associated with reduced cognitive decline and the risk of AD [138]. In transgenic AD mice, the positive effects of EVOO in preventing and delaying the onset of AD and declining the severity of its symptoms have been reported [139]. However, in spite of the reported biological effects, it has not been clear how efficiently HT acts against AD progression. Six-month HT administration to three-month-old female transgenic APP/PS1 mice at 5 mg/kg/day improved the electroencephalographic activity and cognitive function and reduced mitochondrial oxidative stress and neuroinflammation [140]. Moreover, HT was shown to dose-dependently inhibit toxicity of α-synuclein aggregation in PD [141] and HT and OLE improved spatial working memory and energetic metabolism in the brain of aged mice [142]. Notably, HT efficiently neutralizes free radicals and protects biomolecules from ROS-induced oxidative damage. In this regard, HT activates the Nrf2–antioxidant response element (ARE) pathway, leading to the activation of phase II detoxifying enzymes and the protection of dopaminergic neurons exposed to hydrogen peroxide or to 6-hydroxydopamine [20,143]. In addition, HT improved mitochondrial function and induced phase II antioxidative enzymes, which decreased oxidative stress in the brain of db/db mice [144]. Recently, it has also been shown that OLE improves mitochondrial performance through activation of the Nrf2-mediated signaling pathway protecting the hypothalamus from oxidative stress [145]. In conclusion, HT is being considered with interest for possible use in pharmacological mitigation of neurodegenerative processes as a potent antioxidant and a novel small molecule that can upregulate the Nrf2-ARE pathway.

Interestingly, plant polyphenols upregulate the vitagene signaling pathway that represents a potential therapeutic target in the crosstalk of inflammatory response process and oxidative stress in neurodegeneration. Accordingly, recent in vitro and in vivo studies have indicated that HT attenuates the NLRP3 inflammasome pathway by reducing pro-inflammatory interleukin (IL)-1β and IL-18 cytokine levels, oxidative stress, neuronal apoptosis, via activation of the Nrf-2/HO-1 signaling pathway and suppression of NF-κB [146,147,148]. Previous research revealed that HT and OLE activate the signaling pathway from Sirt1, an important member of the vitagene family that catalyzes the deacetylation of various substrates, by utilizing nicotinamide (NAD^+^) as a substrate, to restore adaptive homeostasis under stress conditions [149]. By regulating cellular redox homeostasis, Sirt1 plays a critical role in neuron survival, insulin sensitivity, mitochondrial biogenesis, neurogenesis, and inflammation [114]. Sirt1 has been shown to be essential for synaptic plasticity, cognitive functions [150], modulation of learning, and the preservation of memory processes that deteriorate during aging, in the brain [151]. Recently, in vitro and in vivo studies suggest that HT and OLE inhibit the inflammatory response through different pathways regulated by several members of the sirtuin family (e.g., Sirt1, Sirt2, Sirt6) [140,152,153,154,155,156].

The thioredoxin system (Trx/TrxR) is a meaningful thiol/disulphide redox controller ensuring the cellular redox homeostasis [157,158]. In addition to the regulation of the expression of the encoding genes, TrxR activity is also regulated post-translationally by the thioredoxin inhibitory protein (TxNIP). In this context, a recent study has reported that HT induces neuroprotection and cellular antioxidant defenses via upregulation of the Keap1-Nrf2-TRXR1 pathway [159]. Sulforaphane is an herbal isothiocyanate enriched in cruciferous vegetables obtained in high concentrations from broccoli seeds and sprouts; myrosinase, an enzyme segregated in plant cells and released when the latter are masticated and ingested, produces sulphoraphane by glucoraphanin hydrolysis. This molecule has been shown to be active by fostering cellular defenses against a broad spectrum of cellular stresses. Evidence has shown that sulforaphane in the CNS activates Hsps and related mechanisms central to multiple cellular processes, including synaptic transmission, and improves cortical connectivity [160]. In addition, sulforaphane can also decrease STAT-1 and NLRP3 inflammasome activation by the Nrf2/HO-1 signaling pathway in human microglia-like THP-1 cells [161]. Finally, a recent in vivo study showed that sulforaphane modulates Hsp70 upregulating C-terminus of Hsp70-interacting protein (CHIP) and has the potential to reduce the deposition of Aβ and tau in a mouse models of AD [162].

Several studies have reported the antioxidant and anti-inflammatory properties of resveratrol, presently under clinical trial against neurodegenerative disorders. Recent compelling evidence indicated that resveratrol at micromolar concentrations can effectively scavenge free radicals and is an importantallosteric activator of the vitagene signaling pathway. In addition, resveratrol is involved in the neuroprotective mechanisms by increasing the Nrf2 pathway and reducing NF-κB activity, and therefore exerts a positive effect against a neuroinflammatory state and counteracts the progression of brain aging [163]. Some studies have also shown that resveratrol improves the learning and memory deficit in neurodegenerative disorders and, through its antioxidant activity, protects against memory decline in AD [164]. One of the major mechanisms of resveratrol as neuroprotective molecule is the activation of Sirt1 that is expressed in the adult mammalian brain, predominantly in neurons [165]. Moreover, resveratrol upregulates the Sirt1 pathway, thus preventing Aβ-induced microglial death and contributing to improve cognitive function [166] and induces the SIRT-1 and TRX signaling pathways with reduction of Aβ-stimulated NF-κB signaling, that contributes to its strong neuroprotection in AD [167,168,169,170].

Some preclinical studies have also suggested a positive role of curcumin as an adjuvant therapeutic strategy in free radical-based disorders, particularly neurodegenerative disorders. Some studies have reported the key role played by HO-1 as a target for neuroprotection by curcumin [117,171]. Several studies showed that resveratrol and curcumin increase HO-1 expression in PC12 cells and endothelial cells, among other cell lines [168,170,172]. Moreover, it has been suggested that curcumin inhibits IL-1β secretion and NLRP3 inflammasome activation in macrophages, reduces soluble tau oligomers, and improves cognition by increasing HSP70, HSP90, and HSC70, in aged human tau transgenic mice [173,174].

Taken together, all these data indicate that the activation of stress responsive mechanisms following moderate and chronic consumption of low doses of plant polyphenols induces the vitagene signaling pathway, thus activating antioxidant and neuroprotective cascades that could be effective to prevent neuroinflammation in aging-associated cognitive decline, and thereby improves health-life and longevity in animals and humans (Table 2).

### 5.2. Metabolic Homeostasis

Traditionally, the main aim of nutrition is to provide correct amounts of dietary principles to prevent and, when needed, to treat, nutritional deficiencies. However, when nutrition is inadequate or excessive, the body faces the problem of controlling the amount of nutrients absorbed and stored, with the ensuing emergence of diet-associated pathologies, including MetS. The latter is a complex of symptoms and pathological conditions appearing in people over 65, particularly in females [175], induced by insulin resistance; MetS includes T2DM, cardiovascular diseases, obesity, non-alcoholic liver steatosis, and cancer [176]. Growing evidence indicates a significant reduction of the incidence of T2DM and MetS by the intake of plant polyphenols [177], particularly those found in the EVOO [57,178]. MetS treatment include lifestyle modifications such as physical activity, weight control, and intake of plant food products, such as whole grains, berries, fruits, and vegetables, all known to be sources of many polyphenols [179,180].

The regulation of blood glucose levels is dependent on the liver, in coordination with muscle and adipose tissue. Particularly, in the postprandial state, the liver involves several enzymes such as glucokinase and glycogen synthase to store glucose via the glycogenesis pathway. Moreover, the produced glucose by the liver, via glycogenolysis or gluconeogenesis pathways, depends by pyruvate carboxylase, phosphoenolpyruvate carboxykinase, fructose-1,6-bisphosphatase, and glucose-6-phosphatase activities [181]. In abnormal conditions, the increased post-prandial Apo-B48 levels correlate with the blood glucose levels and lipid profile [58], promoting and/or worsening the atherosclerotic process [182]. A correlation between oxidative stress and incidence of cardiovascular events was observed in diabetic vs. non-diabetic patients with post-prandial glycaemia [183]. Indeed, a recent study in patients with diabetes mellitus (DM) reported a correlation between a high-fat meal and enhanced circulating levels of gut-derived bacteria lipopolysaccharide (LPS) [184]; this modification may represent an important activator step for systemic post-prandial oxidative stress as LPS is responsible for activation of Nox2, a most important ROS producer [185]; overall, data obtained in animal models and in human studies highlight the beneficial effects on postprandial blood glucose levels of natural polyphenols derived from food fractions and beverages. It has been observed that OLE and HT in diabetic rats modulate activities of hepatic antioxidant enzymes, superoxide dismutase, and catalase, attenuating the oxidative stress associated with diabetes [186]. Other data reported that EVOO consumption is associated with down-regulation of Nox-2-derived oxidative stress [187], indicating its ability to mitigate post-prandial oxidative stress via lowering post-prandial LPS also in patients with impaired fasting glucose [188]. Another study highlighted the different pattern of postprandial glycaemia induced by consumption of cranberry juice sweetened with high-fructose corn syrup with respect to comparable amount of a sweetener in water [189]. Finally, a very recent study carried out on stroke-affected mice fed with HT-supplemented diet showed a remarkable recovery of motor and cognitive functions, and of magnetic resonance imaging parameters, together with improvement in neuroinflammation and neurogenesis with respect to controls mice fed with normal diet [190].

**Figure 4:**
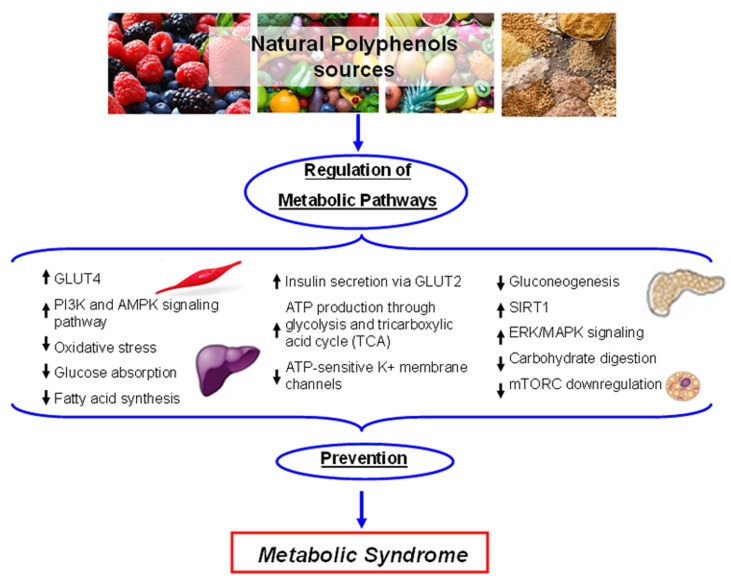
Schematic representation of the main metabolic pathways influenced by plant polyphenols. Activated pathways: **↑**; Inactivated pathways: **↓**.

Plant polyphenols influence glucose metabolism through several mechanisms such as reduction of intestine carbohydrate digestion and glucose absorption, stimulation of pancreatic β-cells to secret insulin, modulation of liver glucose release, activation of insulin-sensitive tissues in terms of insulin receptors, and glucose uptake. In this context, different polyphenols have been shown to inhibit the enzymes involved in digestion to glucose of dietary carbohydrates, α-amylase and α-glucosidase [180], reducing the rate of glucose release and absorption, thus suppressing post-prandial hyperglycemia [191,192]. The polyphenols for which these inhibitory effects have been reported include quercetin, myricetin, luteolin, EGCG theaflavin, and resveratrol [193,194,195]. Moreover, several flavonoids and phenolic acids, such as tannic acids, quercetin, and myricetin, have been reported to inhibit the sodium-dependent SGLT1 and the sodium-independent GLUT2 glucose transporters in cultured intestinal cells [196,197]. Furthermore, it has been reported that in vitro catechins of green tea [198], procyanidins derived from grape seed [199], bitter melon [200], phenols of EVOO [11], and black soybean [201] act on a GLUT4-mediated process to enhance insulin-mediated glucose uptake. Otherwise, data obtained from T2DM mice model demonstrated that black soybean seeds, rich in anthocyanins and procyanidins, reduced glucose levels and increased insulin sensitivity activating the PI3K and 5′-adenosine monophosphate-activated protein kinase (AMPK) pathways in the skeletal muscle and liver [202]. Moreover, AMPK activation was also observed in OLE-treated C2C12 cells and in high-fat-diet mice and resulted as being correlated with the up-regulation of GLUT4 in skeletal muscle and with the down-regulation of liver gluconeogenesis [203,204,205]. The increased GLUT4 expression induced by OLE treatment, particularly in association with insulin, reduced oxidative stress and enhanced glucose consumption, improving insulin sensitivity [206]. Differently, in pancreatic β-cells, the glucose-stimulated insulin secretion resulted from glucose entrance via GLUT2, glycolysis, and tricarboxylic acid cycle with increased ATP production and inactivation of ATP-sensitive K^+^ membrane channels [207]. Treatment with EGCG and buckwheat, the flavonoid rutin, resulted in reduced glucotoxicity to β-cells by activation of insulin receptor substrate 2 (IRS2) and AMPK signaling, and increased ATP levels [208,209]. Other data obtained with the RINmF5 cell model indicated that polyphenols such as quercetin, apigenin, and luteolin inhibit β-cell damage through suppression of NF-κB activity [210]. Moreover, the glucose-stimulated insulin secretion in β-cells was promoted by OLE treatment, but not its moiety HT, by activating ERK/MAPK signaling [211]. Other studies reported that HT reduces the oxidative stress in adipocytes, increasing the activity of AMPK, acetyl CoA carboxylase, hormone- sensitive lipase, and lipase phosphorylation, thus reducing the risk of obesity, a condition that correlates positively with the MetS [209,210] (Figure 4). Many of the reported metabolic and cellular physiologic effects elicited by plant polyphenols share a common upstream mechanism involving AMPK activation/mTORC inhibition. The serine/threonine kinases AMPK and mTORC are key co-ordinators and controllers of many molecular routines including those regulating cell metabolism (anabolism or catabolism) autophagy and proteostasis, cell proliferation, and the redox condition [210,212]. In turn, AMPK and mTORC activities are under control of several factors. AMPK, a regulator of cellular energy homeostasis [213], is triggered by energy stress signalled by reduced ATP and increased AMP levels [211]. AMPK is also activated by phosphorylation of two regulatory subunits by several kinases, including the energy stress-sensitive LKB [214], PKA [215], and the CAMM-GSK3β axis [216] as well as by other factors, including PPARα, associated with lipid metabolism [217] and hypoxia [218]. AMPK triggers the expression of antioxidant enzymes, notably HO-1, through Nrf2 [219,220], and improves the control of proteostasis [221] through direct activation of FOXO3 [222] and ULK1 [223] and by indirect activation of TFEB [224]; AMPK activation also improves both glucose metabolism by favoring GLUT4 translocation to the membrane [225,226] and lipid metabolism through PPARα. Finally, AMPK activation results in inhibition of mTORC following both direct phosphorylation and phosphorylation of the mTORC controller TSC [227].

Some of the mechanisms reported above are also triggered by plant polyphenols. In particular, it has been reported that OLE increases intracellular free Ca^2+^ levels from the internal stores with ensuing activation of the calcium-CAMMK-GSK3β pathway [216] and reduces oxidative stress through AMPK-dependent activation of HO-1 [219], whereas in aged rats, resveratrol protects against high-fat diet-induced muscle atrophy counteracting PKA/LKB1/AMPK-mediated mitochondrial dysfunction and oxidative stress [215]. Finally, plant polyphenols including quercetin, resveratrol, and catechins activate SIRT1, a class-3 histone deacetylase involved in several aging-related pathologies, including neurodegeneration [228]. Recent data with TgCRND1 mice indicate that SIRT1 is activated by OLE, with concomitant inhibition of PARP1 in a crosstalk affecting apoptosis and autophagy [40]. It is remarkable that some of these effects by several plant polyphenols are shared with the well-known antidiabetic drug metformin [229]. These molecules have different ways of accomplishing their functions and work in different cell types; however, taken together, the vast amount of research and preclinical data provide a convincing, yet still incomplete, explanation of the cellular protection by these substances that underlies their hormetic behaviour (see Figure 4).

### 5.3. Proteostasis

Proteostasis is a key biological process under strict cellular control by maintaining the equilibrium between protein synthesis and protein degradation by different protein degradation machineries (the chaperone–ubiquitin–proteasome and various forms of autophagy), whose homeostasis is needed for a healthy life span. The proteostasis network needs the coordinated action of both chaperones and the ubiquitin–proteasome system (UPS), and several types of autophagy, notably macro-autophagy and chaperone-mediated autophagy. Molecular chaperones, in particular the heat shock proteins (HSPs), are the prime line of defense to counteract protein misfolding and the ensuing protein deposition; they recognize hydrophobic regions of proteins and promote either their refolding or their transfer to the UPS or autophagy systems [230]. The small HSPs are ATP-independent chaperones, a particular class of HSPs that bind misfolded and aggregation-prone proteins, avoiding undesired intermolecular interactions and preventing their aggregation with ensuing loss of function [231,232]. The UPS is localized both in the cytoplasm and in the nuclei; it provides rapid degradation of proteins following their polyubiquitination and subsequent delivery to the proteasome. The misfolded proteins of the secretory pathway are managed by the endoplasmic reticulum-associated protein degradation (ERAD) pathway.

Differently from the UPS and the ERAD pathways, autophagy, a self-digestive process triggered by nutrient deprivation, aging, and other stressful conditions including energy shortage, heath shock, and oxidative stress, is important for long-lived proteins and organelles clearance. It requires the participation of a number of proteins encoded by autophagy-related genes (Atg) that are necessary to the formation of autophagosomes, double membrane vesicles, where the cargo material is engulfed and their subsequent delivery to lysosomes for degradation [233]. In addition to the proteostasis network, the cells can also alleviate intracellular accumulation of misfolded proteins through a selective secretion of damaged proteins and RNAs into vesicles of endosomal origin, called exosomes. Several evidences have stressed a crosstalk between the autophagic pathway and the exososomes [234].

The proteostasis network can decline permanently or transiently following development, aging, and exposure to environmental stress, and this decline may contribute to adult-onset proteotoxic disorders and their progression. In particular, a continuous reduction of the efficiency of the proteostasis network may induce pathological aging with accumulation of abnormal proteins, a common feature of many oncological, neurodegenerative, metabolic, and cardiovascular disorders. Proteostasis deregulation at the level of the ER is one of the major contributors to aging and cancer as well [235]. Indeed, harmful stimuli, such as oxidative stress and disruptions of the secretory process may contribute to deposition of unfolded or misfolded proteins at the ER lumen, thus upregolating the endoplasmic reticuluym (ER) stress response [236]. Finally, cancer cells accumulate genetic changes resulting in the presence of mutated proteins; accordingly, most cancer cells are closely dependent on the proteostasis network for survival.

Scientific evidence shows that natural molecules, such as polyphenols, exhibit healthy and protective effects as consequence of their ability not only to maintain the proper oxidant/antioxidant balance in cells, but also for their anti-inflammatory power together with their ability to control the activity of signaling pathways responsible with cell survival, proliferation, and migration. Moreover, the pleiotropic anti-amyloid properties of the polyphenols indicate that these molecules may be useful for treatment of several amyloidosis, notably T2DM and several neurodegenerative diseases. Indeed, many plant polyphenols can reduce the aggregation of some proteins/peptides involved in metabolic and neurodegenerative diseases and can directly or indirectly enhance the clearance of misfolded proteins by modulating the activity of the proteostasis network. However, recent data indicate that increased efficiency of proteostasis and stress resistance may also favor cancer progression. The stresses accomplished by cancer cells require active chaperone platforms to limit protein misfolding and aggregation; the latter, in these cells, may result from high levels of protein synthesis and metabolic requests, oxidative stress, growth under hypoxic and acidic conditions, and protein expression in altered stoichiometries resulting from aneuploidy [237]. The modulation of the proteostasis network by small regulatory molecules can provide a previously unexploited and potentially powerful approach to improve proteome balance.

Resveratrol, one of the most investigated plant polyphenols, displays eclectic biochemical behavior; in fact, it acts upstream of several signaling cascades, likely upon binding to membrane receptor(s); actually, potential binding sites were detected in the plasma membrane of neuronal cells and, to a lesser extent, in nuclear and cellular fractions of rat brain homogenates [238]. In addition, resveratrol mimics some aspects of the CR by inhibiting cAMP phosphodiesterase with activation of AMPK, with reduced fat accumulation, increased glucose tolerance, insulin sensitivity, mitochondrial biogenesis, and physical endurance. Besides its antioxidant activity [239], resveratrol reduces both intracellular and secreted Aβ in cell culture by activating chaperones such as Hsp70 and by inducing proteasome-dependent Aβ degradation [240]. In addition, resveratrol activates SIRT-1, increases AMPK activity, thus reducing Aβ-induced death signals, such as NF-κB signaling and p53 activity [240] and triggering the degradation of Aβ aggregates by autophagy [239]. The anti-cancer property of resveratrol occurs by inducing caspase 8- and caspase 3-dependent apoptosis via ROS-induced autophagy in human colon cancer cells [241]. In androgen-independent prostate cancer cells, resveratrol induces autophagy-mediated cell death through regulation of store-operated Ca^2+^ entry (SOCE) mechanisms, and down-regulation of stromal interaction molecule 1 (STIM1) expression. In addition, resveratrol triggers ER stress by depleting the pool of ER calcium [242]. Resveratrol has also been shown to decrease the proliferation of breast cancer stem-like cells by inhibiting Wnt/β-catenin signaling pathway [243]. Finally, resveratrol has been shown to act as an inhibitor of global protein synthesis in the H4-II-E rat hepatoma cell line. This effect was reached through modulation of mTOR self-phosphorylation and hence of mTOR-dependent and mTOR-independent signaling by reduced formation of the eIF4F translation initiation complex and increased phosphorylation of eIF2α with inhibition of translation [244]. Other findings in renal tissues of rats fed with resveratrol after unilateral uretral obstruction point to the reduced expression of eIF2α and the ensuing reduced levels of ATF4 [245].

Other plant polyphenols have been investigated for their ability to control the proteostasis system at several levels. Curcumin displays several beneficial effects in different in vivo models of aging, ischemia, trauma, and neurodegeneration. In in vivo models of proteinopathies, curcumin reduces plaque burden and improves cognitive function [246]. In addition, curcumin has been shown to induce the expression of Hsp genes and the nuclear translocation of Hsf1, a master regulator of Hsp expression [247]. Green tea catechins show conformational similarities to chaperones and a chaperone-like activity [248]. Phenolic and flavonoid components present in bee pollen activate the chymotrypsin-like activity of the proteasome in HFL-1 human embryonic fibroblasts [249]. OLE displays neuroprotection by increasing proteasome activity, and extending the life span of human IMR90 and WI-38 embryonic fibroblasts [250]. The administration of OLE to the *C. elegans* C2006 strain expressing Aβ42 in muscle cells induced a significant reduction of amyloid plaque and toxic oligomer formation, with reduction of the extent of paralysis and increased lifespan [132]. The dietary administration of OLE also improved the cognitive performance of the TgCRND8 mouse model of Aβ deposition resulting from reduced plaque deposits, increased microglia migration to the plaques for phagocytosis, and an intense autophagic reaction followed by modulation of the mTOR/AMPK pathways [14,40]. Recent data with cardiomyocytes indicate that OLE counteracts MAO-A cytotoxicity and that this effect results from restoration of restoration of the defective autophagic flux, as indicated by the increase of autolysosomes, indicative of autophagosome–lysosome fusion. Interestingly, autophagy induction involved nuclear translocation and activation of the master gene for lysosomal biogenesis TFEB, suggesting a role of OLE as a TFEB activator [251].

Quercetin has been shown to have neuroprotective effect in various in vitro and in vivo systems. In malignant mesothelioma, quercetin inhibits cell growth and increases the levels of Nrf2, with the transcriptional activation of genes involved in the control of the cellular redox status [252]. In addition, quercetin may induce the expression of chaperones and of proteasome subunits through the Nrf2 pathway [253]. In primary effusion B cell lymphoma, quercetin induces apoptosis and autophagy by inhibiting the PI3K/AKT/mTOR and STAT3 signaling pathways, with the ensuing down-regulation of the expression of pro-survival cellular proteins such as c-FLIP, cyclin D1, and cMyc [254].

## 6. Epigenetic Implications

**Figure 5:**
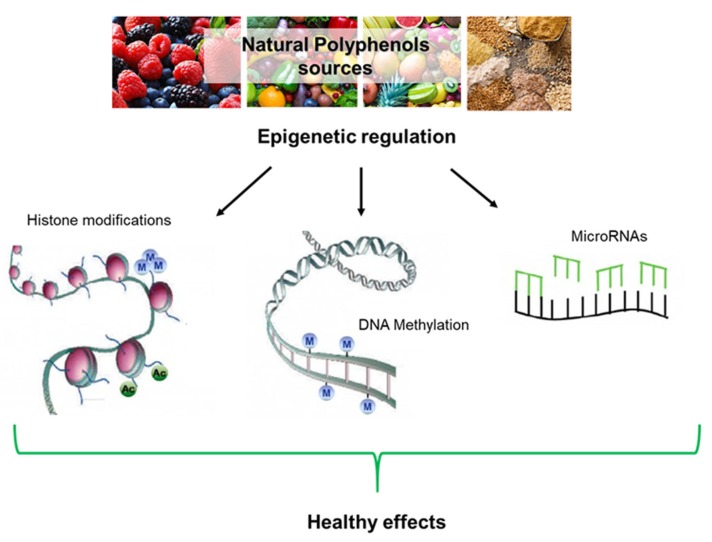
A summary of the epigenetic modifications mediated by plant polyphenols.

The pivotal mechanisms of genetic regulation, that induce or repress the expression of genes, include genetic mutations and epigenetic modifications, the last include DNA methylation, histone acetylation, methylation or phosphorylation, and RNA interference (RNAi). When aberrant, these modifications influence the onset and worsening of diseases and these alterations are considered as essential hallmarks for distinct types of cancer, neurodegenerative diseases, metabolic disorders, bone, and skeletal diseases [2]. In principle, drugs are able to reverse epigenetic defects on the contrary to genetic alterations [255]. In this regard, promising results for cancer treatment have been obtained from two groups of drugs with inhibitory activities on DNA methyl transferase (DNMT) and histone deacethylase (HDAC) [256]. Recently, most studies demonstrated epigenetic modifications induced by natural polyphenols treatment, allowing the introduction of the term “epigenetic diet” [257,258,259]. As an example, several polyphenols, including curcumin, resveratrol, and catechins, are reported to act on the NF-κB expression and chromatin remodeling through modulation of HDACs and DNMTs activity [260] (Figure 5). Below, several studies supporting plant polyphenols effects on epigenetic are reported with possible clues for treatment of specific diseases.

### 6.1. DNA Methylation

The presence or the absence of 5-methylcytosine define DNA methylated regions of DNA template. The methylation on cytosine residues depends on DNMTs or 10 or 11 translocation methyl-cytosine dioxygenases (TETs) activity. In this way, the gene expression is regulated by DNA methylation in a region-specific manner, and occurs predominantly at cytosines preceding a guanine nucleotide (CpG sites). Usually, DNA hypermethylation occurs at promoter CpG islands and is a major epigenetic mechanism to silence gene expressions [261]. However, at present, the methylation process must establish a relationship between of intronic, exonic, or untranslated regions and the expression pattern of the encoded proteins [262]. The acetylation and methylation mechanisms reverse the epigenetic modifications of genes that include chemical modification, e.g., the methylation at CpG dinucleotides, and the ensuing transcriptional activation of histone complexes [263]. The DNA methylation of some developmental genes is a highly controlled mechanism that involved the NAD-dependent deacetylase SIRT1 that selectively prevents abnormal methylation process [264]. Sirtuins, NAD^+^-dependent enzymes, are considered amongst the most promising anti-aging targets, leading to define these deacetylases as “the enzymes of youth”. Sirtuins depend on AMPK via stimulation of nicotinamide phosphoribosyltransferase (NAMPT), an enzyme involved in NAD^+^ synthesis that regulates cellular metabolism in several mammalian organs and tissues [265] and whose inhibition worsens diet-induced hepatic steatosis in mice [266].

Recent studies have provided a lot of data to support that plant polyphenols and other phytochemicals remarkable impact on DNA methylation by causing direct or indirect modifications of DNMTs levels and activity. For example, genistein, one of the phytoestrogens found in soybeans, reduces DNMT activity, forming a complex with it, and activates tumor suppressor genes, with possible significance for cancer prevention and therapy [267]. Other data indicate that resveratrol activates both the PGC1α/SIRT1/AMPK axis and DNMTs, with beneficial effects for cancer prevention and treatment [268]. Similar results have been reported for quercetin [269] and EVOO polyphenols; the latter were shown to activate the Sirt1 path, with possible relevance for AD prevention/treatment [40]. Furthermore, studies performed on esophageal, oral, skin, regulatory T-cells, lung, breast, and prostate cancer cells reported alterations induced by bioactive compounds from green tea, including EGCG, on DNMT activity in studies performed on esophageal, oral, skin, regulatory T-cells, lung, breast, and prostate cancer cells [270]. In detail, Fang et al. suggested an EGCG inhibitory effect on DNMT and reactivation of methylation-silenced genes in human colon, esophageal, and prostate cancer cells [271]. Finally, it appears that the inhibition of DNMT is a chemopreventive effect on cancer cell growth by genistein, suggesting its use as anti-neoplastic drug in some malignancies [272].

### 6.2. Histone Modifications

The primary components of the epigenome are the post-translational modifications (PTMs) of histones often leading to charge-induced changes in the nucleosome, with major influence on gene expression. Histone PTMs also regulate several biological processes by chemical alterations of chromatin and the ensuing modulation of gene expression that contributes significantly to aging-associated dysfunctions and to tumor development and carcinogenesis [273]. Histone modifications can result from the activity of various enzymes, including histone acetyltransferases (HATs), histone deacetylases (HDACs), histone methyltransferases (HMTs), and histone demethylases (HDMs). The histone acetylation is a process involved in the regulation of different cellular processes such as transcription, gene silencing, apoptosis, DNA repair, and cell differentiation [274]. Differently, HDACs catalyze histone de-acetylation, signal transduction, apoptosis, and cell growth [275].

As modulators of histone modifications, many dietary polyphenols hold promise to be effective for prevention and therapy of neurodegeneration and some cancers. For example, the inhibition of HDAC activity is due to the interaction between sulforaphane complexes with the active sites of HDACs [276]. EGCG not only modifies DNA methylation, but also acts as a histone modifier, interfering with the HDAC and HAT activities [277]. Quercetin behaves as an HAT inhibitor and suppresses HAT activity in human endothelial cells [278]; moreover, a recent study suggests that it can also inhibit HDAC [279]. Recently, it has been reported that the co-treatment with suberoylanilidehydroxamic acid (SAHA), an HDAC inhibitor, and naringenin, a flavone present in citrus, synergistically induces cytotoxic effects in neuroblastoma with respect to non-malignant neuronal cells [280]. Genistein is the polyphenol with the highest ability to modify histone activity compared to other isoflavones; indeed, it was reported an increased HATs and decreased HDACs activity after treatment with this compound of precancerous and cancer breast cells [281,282,283]. Otherwise, clinical benefits in cancer cells have been obtained after curcumin treatment, resulting in down-regulation of HATs activity [284].

Recent data indicate that the protective effects of OLE in the diet of a Tg murine model of Aβ deposition could be traced bask, at least in part, to the increased acetylation of histone 3 and 4, that matched the reduced expression of HDAC-2 [40], whose levels are increased in the AD brain [285,286] and of glutaminyl cyclase (GC), the enzyme responsible for the generation of pE3Aβ, the most aggregating pyroglutamylated derivative of the Aβ_1–42_ peptide in AD [287]. Interestingly, GC is significantly increased in the brain of AD patients [288]. Similar epigenetic effects have been reported in cancer cells [289]. Oleacein, another EVOO polyphenol, also induces a strong increase of histone and α-acetyl-tubulin acetylation [290].

### 6.3. Noncoding RNAs

MicroRNAs (miRNAs) are small non-coding RNA molecules, 20–22 nucleotides in length, organized in a single-strand structure, with the function of RNA silencing and post-transcriptional regulation of gene expression [291]. miRNAs significantly participate in epigenetic regulation; in fact, they perform catalytic functions related to RNA splicing and significantly contribute to post-translational gene regulations. Accordingly, miRNAs regulate many cellular processes, including cell proliferation, apoptosis, cell differentiation, and any alterations of their expression correlate with different disease progression. Many studies support a direct association between alterations of miRNAs expression and cancer [292]. Indeed, bladder, lung, and breast cancer onset correlates with different mechanisms of regulation of miRNAs expression including chromosomal abnormalities, single nucleotide polymorphisms (SNPs), mutations in primary transcripts [293], altered activity of transcription factors, such as the miR-17-92 cluster, and modifications in the miR-34 family such as consequence of p53 activation [294,295].

Recently, EGCG has been found to decrease the expression of oncogenic miRNAs (miR-92, miR-93, and miR-106b) and to increase the expression of tumor-suppressor miRNAs (miR-7-1, miR-34a, and miR-99a) in human cancer cells [296,297]. Another study in apolipoprotein E-deficient mice reported that the treatment with one of nine different polyphenols including hesperidin and naringenin affected the expression of five miRNA in vivo [298]. Curcumin also regulates pancreatic cancer symptoms by up-regulation of miR-22 and down-regulation of miR-199a [299]. However, the potential use of curcumin and other polyphenols against cancer cells trough modulation of miRNAs is very prominent, but at the same time, limited because of the reduced bioavailability of these substances. In spite of these limitations, the data currently available contribute to explaining the healthy effects of flavonones [298].

## 7. Conclusions

A growing number of population surveys and clinical trials increasingly support the use of plant polyphenols, possibly in association with more specific drugs, to prevent and/or to treat several aging-associated pathologies [300]. However, in a number of cases, contradictory results have been reported possibly due to different experimental settings in terms of dose, length of treatment, clinical conditions of the enrolled patients at the onset of treatment, and other unknown variables. In fact, the low bioavailability of polyphenols must be taken into account, as well as the fact that the latter could be further affected by specific physiological features of different patients, also taking into account the role played by the gut microbiota. Moreover, data are still lacking that properly focus the pharmacodynamics and pharmacokinetics of these molecules in different clinical settings. However, the overall picture that emerges from the body of the data currently available strongly suggests the nutraceutical value of these substances, yet with differences from each other.

The positive results arising from population surveys and clinical trials are increasingly supported at the molecular/cellular/tissue level by the large body of studies carried out recently that have, at least in part, unraveled the molecular basis and the cellular correlates of the healthy effects recorded at the organismal level. What emerges from these studies is the ability of plant polyphenolsthese to interact with many regulatory molecules modulating a number of signaling paths that maintain proteostasis as well as the redox and metabolic homeostasis or restore any disequilibrium of these conditions. In this sense, the data on the modulation of several epigenetic modifications by some of these molecules, although still limited, are of importance, providing clues as to how plant polyphenols can produce so many heterogeneous effects at the cellular level, integrating and extending the hormetic theory.

In conclusion, the rising interest in the nutraceutical/pharmacological exploitment of plant polyphenols or their molecular scaffolds holds promise that in the near future, the knowledge of the molecular/cellular determinants of the beneficial effects of these molecules, together with their pharmacokinetics and pharmacodynamics, will increase. It is also expected that results from more extended and convincing clinical trials will be reported, better focusing on benefits and, possibly, caveats associated with the use of these molecules or their chemical derivatives. Such increased information, provided it will further confirm the potential of plant polyphenols in the prevention/treatment of metabolic-, aging-, or lifestyle-associated pathologies presently without resolutive therapies, will allow a more general use of these molecules as an important tool to prevent or to reduce the incidence of these increasingly widespread pathologies, ensuring safer aging.

## Figures and Tables

**Table 1:** A list of relevant population studies and clinical trials carried out with plant polyphenols or polyphenol-enriched foods.

Name/Number	N° People	Age	Type of Study	Treatment	Pathology	Ref
Seven Countries Study	12,763	40–59	Population, longitudinal	Traditional eating	CVD	[46]
Lyon Diet Heart Study	605	Recur.	Random. sec. prevent. trial	MD	Myo infarct.	[47]
Three-City Study	9077	>65	Multi-center cohort study	Olive oil	Cognition	[48]
PREDIMEDNAVARRA	1877/522	74 (mean)	Observational study	MD/oil or nuts	CVD risk/Cognition	[49,50,51,52]
PREDIMED	447	55–80	Dietary-intervention trial	Polyphenol intake	CVD profile/cognition	[51]
Spanish people	4572	>18	Cross-sect. popul. Study	Olive/sunflower oil	CVD risk factors	[57]
Nurses’ Health Study	59,930	35–65	Longitud. Associate. study	Olive oil	Risk of T2DM	[58]
NHS II	85,157	26–45	Longitud. Associate. study	Olive oil	Risk of T2DM	[58]
Not specified	25	unknown	Cross-over study	MD with EVOO	Gluc./LDL chol	[59]
EUROLIVE	200	various	Randomized crossover trial	EVOO	Ox.stress mark./Chol.	[60,61,62]
PREDIMED Study	7447	55–80	Multicenter trial	MD/EVOO or nuts	CVD risk	[63]
Not specified	20	various	Double-blinded rand. Cross.	EVOO	Gene express.	[64]
Not specified	20	various	Randomized crossover	Olive polyphenols	Postprandial infl.	[65]
Not specified	62	65–96	Observational study	EVOO	Antioxidant status	[69]
Not specified	410	elderly	Random. double-blind trial	Ginkgo extract	Dementia	[68]
MedLey	166	>65	Random. control. Int. trial	MD	Cognition decay	[72]
ISRCTN35739639	447	66.9 (mean)	Randomized clinical trial	MD/EVOO or nuts	Cognition	[74]
Not specified	20	>50	Random. double-blind clin. trial	4 g Curcumin	Cognition	[76]
Not specified	36	aged	Random. double-blind clin. trial	2/4 g Curcumin	AD	[77]
Trials with resveratrol	-	various	244 clinical trials+ 27 ongoing	Resveratrol	various	[79,80]
Rockefeller Univ. Hosp.	28	various	Placebo contr. Clinical trial	1g Resveratrol	MetS, ins. Res.	[81]
Not specified	19	various	Observational study	Green tea extracts	ATTR cardiomyop.	[83]
Not specified	-	various	Various	Ginkgo biloba extracts	Various	[84]
SUN Project	22,786	various	Prospective Med. cohort study	Polyphenols	Breast cancer	[86]
SUN Project	22,786	various	Prospective Med. cohort study	Polyphenols	CVD, T2DM, MetS	[97]
Not specified	79	various	Controlled clinical trial	500 mg Olive leaf extract	T2DM	[89]
Not specified	46	46.4 (mean)	Randomized crossover trial	Olive leaf extract	T2DM	[90]
Not specified	60	45 (mean)	Randomized contr. Clin. trial	Olive leaf extract	CVD risk	[91]
NCT01479699	18	various	Random., double-blind cross-over	OLE + HT	CVD markers	[92]
Not specified	24	various	Gene/miRNAs expression	High/low polyph. EVOO	MetS	[93]
Not specified	22	various	Random. Control. trial	Polyphenol-rich EVOO	Redox/Met. Stat	[94]

**Table 2:** Polyphenols activity in cellular protective pathways. Activated pathways: **↑**; Inactivated pathways: **↓**.

Polyphenols	Anti-Oxidant	Anti-Inflammatory	Anti-Aggregation
Oleuropein Aglycone	**↑**Sirt1 **↑**Nrf2	**↑**Sirt1, Sirt2, Sirt6	Aβ, Tau, α-Syn
Hydroxytyrosol	**↑**Sirt1 **↑**Nrf-2/HO-1 **↑**Nrf-2-ARE **↑**Keap1-Nrf2-TRXR1	**↑**Sirt1, Sirt2, Sirt6 **↓**NLRP3 inflammasome **↓**IL-1β, IL-18	α-Syn Aβ
Sulforaphane	**↑**Hsp70-CHIP	**↓**NLRP3 inflammasome **↓**STAT1	
Resveratrol	**↑**Nrf-2/HO-1 **↑**TRX **↓**Nf-κB	**↑**Sirt1	Aβ, Tau
Curcumin	**↑**HO-1 **↑**Hsp70 **↑**Hsp90 **↑**Hsc70	**↓**IL-1β **↓**NLRP3	Aβ, Tau
